# Monofluoromethylation of *N*-Heterocyclic Compounds

**DOI:** 10.3390/ijms242417593

**Published:** 2023-12-18

**Authors:** Mikhail Yu. Moskalik

**Affiliations:** A.E. Favorsky Irkutsk Institute of Chemistry, Siberian Division of the Russian Academy of Sciences, 1 Favorsky Street, 664033 Irkutsk, Russia; moskalik@irioch.irk.ru

**Keywords:** fluorine, fluoromethylation, nitrogen heterocyles, fluorination, pyrrolidines, imidazoles, indoles, pyridines, quinalines, pyridazines

## Abstract

The review focuses on recent advances in the methodologies for the formation or introduction of the CH_2_F moiety in *N*-heterocyclic substrates over the past 5 years. The monofluoromethyl group is one of the most versatile fluorinated groups used to modify the properties of molecules in synthetic medical chemistry. The review summarizes two strategies for the monofluoromethylation of *N*-containing heterocycles: direct monofluoromethylation with simple XCH_2_F sources (for example, ICH_2_F) and the assembly of *N*-heterocyclic structures from CH_2_F-containing substrates. The review describes the monofluoromethylation of pharmaceutically important three-, five- and six-membered *N*-heterocycles: pyrrolidines, pyrroles, indoles, imidazoles, triazoles, benzothiazoles, carbazoles, indazoles, pyrazoles, oxazoles, piperidines, morpholines, pyridines, quinolines and pyridazines. Assembling of 6-fluoromethylphenanthridine, 5-fluoromethyl-2-oxazolines, C5-monofluorinated isoxazoline *N*-oxides, and α-fluoromethyl-α-trifluoromethylaziridines is also shown. Fluoriodo-, fluorchloro- and fluorbromomethane, FCH_2_SO_2_Cl, monofluoromethyl(aryl)sulfoniummethylides, monofluoromethyl sulfides, (fluoromethyl)triphenylphosphonium iodide and 2-fluoroacetic acid are the main fluoromethylating reagents in recent works. The replacement of atoms and entire functional groups with a fluorine atom(s) leads to a change and often improvement in activity, chemical or biostability, and pharmacokinetic properties. The monofluoromethyl group is a bioisoster of -CH_3_, -CH_2_OH, -CH_2_NH_2_, -CH_2_CH_3_, -CH_2_NO_2_ and -CH_2_SH moieties. Bioisosteric replacement with the CH_2_F group is both an interesting task for organic synthesis and a pathway to modify drugs, agrochemicals and useful intermediates.

## 1. Introduction

Heterocyclic cores are key building blocks for drug design and essential subunits presented in natural products, such as vitamins, hormones and antibiotics. Moreover, 85% of bioactive compounds include heterocyclic rings, and most of them are nitrogen heterocycles [1,2]. The predominance of *N*-heterocycles in biologically active compounds can be explained using their stability, efficiency, wide possibilities of chemical modification [3,4,5,6] and ability to form hydrogen bonds with DNA [1]. About 60% of small molecule drugs contain *N*-based heterocycles as common architectural cores [1]. Nitrogen-containing heterocycles are of particular importance, for example, in cancer chemotherapy [7]. Therefore, the task of synthesizing and modifying *N*-heterocycles is an important field of synthetic organic chemistry and medicinal chemistry [1,2,8].

The introduction of fluorine atoms is an effective approach to the development of new drugs and agrochemicals [9,10,11,12,13,14,15,16]. Fluorine atoms increase resistance to biological processes and permeability through cell membranes, provide good binding to enzyme targets and increase target selectivity. The properties of fluorine-containing substituents, and accordingly, the properties of the target molecules, as a rule, are significantly different from the properties of other halogenated and, especially, hydrogenated analogs [17]. Thus, organofluorine substrates are interesting objects for study in the fields of theoretical, synthetic, industrial and medicinal chemistry [18]. Fluorine is sterically similar to hydrogen, being at the same time the most electronegative element, which changes both spatial and electronic properties of fluorine-containing molecules compared to non-fluorinated molecular objects. Therefore, the introduction of a fluorine atom does not significantly increase the volume of the molecule, but conformational changes and changes in reactivity are often dramatic. Fluorine-containing substituents are used to modify the basicity or acidity of neighboring functional groups in molecules. Thus, the introduction of fluorine affects the ability to form hydrogen bonds, which are very important weak interactions playing a significant role in the stabilization of biopolymers [19].

Modern organofluorine chemistry studies the synthetic approaches for the modification of functional groups with fluorine to change the physicochemical and pharmacological properties of candidates for drugs, materials and synthetic intermediates. All this emphasizes the great importance of fluorine derivatives in medicinal [15,20,21,22,23,24], agricultural [25,26] and synthetic organic chemistry [24,27,28,29,30,31].

Fluoroalkylation is the main method for the selective and atom-economic synthesis of organic molecules containing fluorine in the side chain. Trifluoromethylation has been known for more than 40 years [32,33,34,35]. Monofluoromethylation is much less studied, and systematic investigations of monofluoromethylation were only recently developed. General information on monofluorination reactions to form monofluoroalkyl derivatives is well presented in the works [36,37,38]. The synthesis of fluoroalkylated sulfur heterocycles was described in a recent review [39].

The monofluoromethyl group is a universal bioisosteric moiety for many groups (-CH_3_, -CH_2_OH, -CH_2_NH_2_, -CH_2_CH_3_, -CH_2_NO_2_ and -CH_2_SH) in biologically active compounds [40,41,42].

There are a number of commonly used monofluoromethylated drugs on the market. For example, Afloqualone is a nicotine antagonist sold as a muscle relaxant. Fluticasone is a widely used anti-inflammatory and analgesic drug prescribed for certain tumors. The inhalational anesthetic Sevoflurane is the most known CH_2_F compound used since 1990 [36]. Some examples of approved or investigational drugs containing CH_2_F groups are presented in Table 1.

## 2. Monofluoromethylation of *N*-Containing Heterocycles

Monofluoromethylation of *N*-containing heterocycles includes two main methodologies: direct monofluoromethylation with simple XCH_2_F sources (for example, ICH_2_F) and assembly of *N*-heterocyclic structures from CH_2_F-containing substrates. CH_2_F-substituted heterocycles are of great importance as targets for the isosteric development of biologically active compounds.

### 2.1. Synthesis of Monofluoromethylated Aziridines

**Scheme 1:**
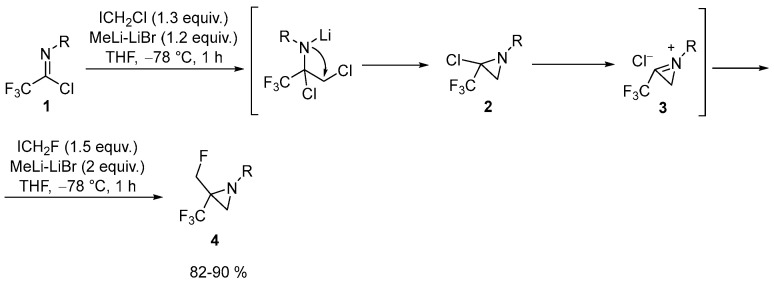
The synthesis of α-fluoromethyl-α-trifluoromethylaziridines **4** via the reaction of trifluoroactimidoyl chloride (TFAIC) **1** and lithium monohalocarbenoids.

The synthesis of α-fluoromethyl-α-trifluoromethylaziridines **4** via the reaction of trifluoroactimidoyl chloride (TFAIC) **1** and lithium monohalocarbenoid was presented in the work [58] (Scheme 1). The reaction is an excellent example of carbenoid assembling of rare fluorinated aziridines **4**.

The reaction proceeds through the aziridination of imine **1** with chloromethyl lithium, followed by treatment with a second equivalent of the fluorine-containing carbenoid of the highly electrophilic azirinium ion **3**, which is formed during the spontaneous removal of the chlorine atom from the intermediate α-chloro-α-CF_3_ aziridine **2** [58]. Fluoriodomethane is a precursor of the second carbenoid–LiCH_2_F, which makes it possible to introduce a fluoromethyl fragment into an aziridine ring to form a number of geminal (fluoromethyl)trifluoromethyl aziridines **4**. The reaction involves the introduction of two halomethylene groups at the sp^2^-carbon atom of the starting imine through sequential treatment with the corresponding iodomethyl halides. The order of carbene precursor usage is important: LiCH_2_Cl is used first as a nucleophile, and LiCH_2_F is used as a second reagent. This order is necessary due to the low nucleophilicity of LiCH_2_F, which cannot initiate the functionalization [58].

**Scheme 2:**
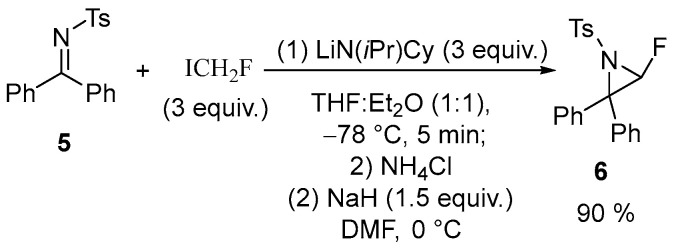
Synthesis of α-fluoroaziridine from *N*-tosyl-substituted diphenyl ketimine and ICH_2_F.

A similar reaction occurs when *N*-tosyl-substituted diphenyl ketamine **5** reacts with ICH_2_F in the presence of LiN(*i*Pr)Cy. The carbenoid and imine **5** yield the hard-to-obtain α-fluoroaziridine **6** in high yield [59] (Scheme 2):

### 2.2. Monofluoromethylation of Five-Membered N-heterocycles

**Scheme 3:**
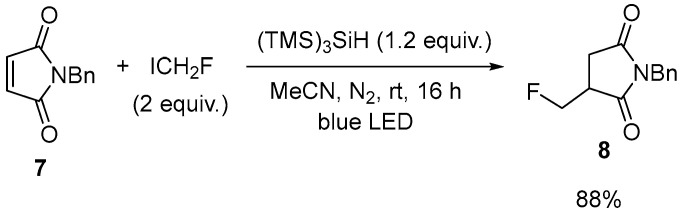
Hydrofluoromethylation of *N*-Benzylmaleimide using ICH_2_F and (TMS)_3_SiH.

*N*-Benzylmaleimide **7** undergoes hydrofluoromethylation with ICH_2_F in the presence of (TMS)_3_SiH upon blue LED activation. The product is formed with a yield of 88% [60] (Scheme 3).

**Scheme 4:**
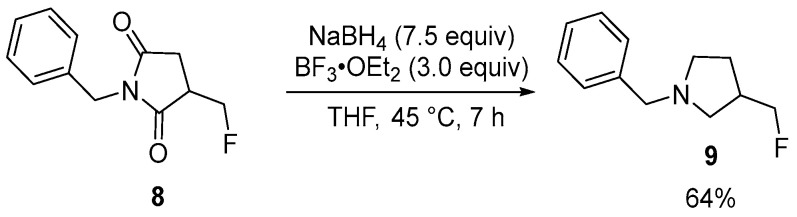
Formation of 3-fluoromethylated *N*-benzylpyrrolidine.

The process of photolytic cleavage of the carbon–iodine bond in the ICH_2_F molecule requires blue LED activation. The fluoromethyl radical combines with the substrate **7** to form an electrophilic carbon-centered radical, which then reacts with tris(trimethylsilyl)silane ((TMS)_3_SiH) to form the hydrofluoromethylated product **8**. The resulting silyl radical (TMS)_3_Si· initiates a chain of radical transformations following the elimination of the iodine radical from fluoriodomethane with the formation of ·CH_2_F and (TMS)_3_Sil· radicals. Based on the obtained product, 3-fluoromethylated *N*-benzylpyrrolidine **9** was synthesized (64% yield) [60] (Scheme 4).

**Scheme 5:**
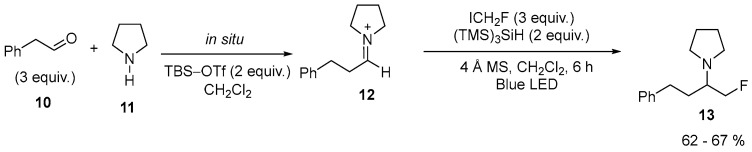
Hydrofluoromethylation of pyrrolidines in the presence of 2-phenylacetaldehyde.

**Scheme 6:**
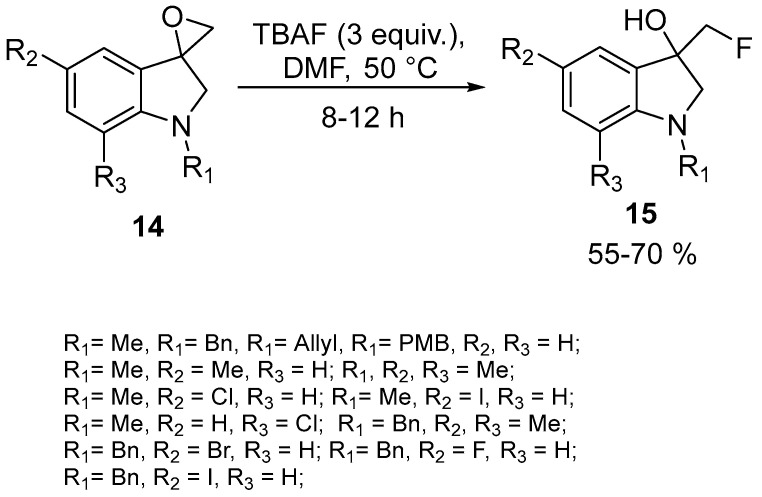
Synthesis of 3-fluoromethylated indolines with nucleophilic ring-opening of oxirane ring in spiro-epoxyoxindoles.

The three-component reaction of 2-phenylacetaldehyde **10**, pyrrolidines **11** and fluoriodomethane under radical activation conditions proceeds through fluoromethylation of the intermediate imine **12** and opens access to monofluorinated simple heterocycles. Product **13** is difficult to synthesize using other methods (Scheme 5) [27]:

The reaction of spiro-epoxyoxindoles **14** and TBAF (tetra-*n*-butylammonium fluoride) in DMF leads to the nucleophilic ring-opening of the oxirane ring, resulting in the formation of 3-fluoromethylated indolines **15** in good yields [61] (Scheme 6):

The method is simple and does not require metal-based catalysts. The approach also makes it possible to reverse regioselectivity in nucleophilic fluorination by changing the nucleophilic fluoride reagents. TBAF allows fluorination in the CH_2_ group. At the same time, Py·(HF)_x_ in CH_2_Cl_2_ opens the oxirane ring at the spiro-carbon atom to form 3-fluoro-3-hydroxymethyloxindole. DMF or CH_2_Cl_2_ in the absence of HF (acidic H-donor), the opening of the epoxide ring proceeds through the S*_N_2* mechanism, which also suggests that both the choice of nucleophilic fluorinating reagents and the choice of the reaction medium play an equally important role in the direction of the regioselectivity of the reaction [61].

**Scheme 7:**
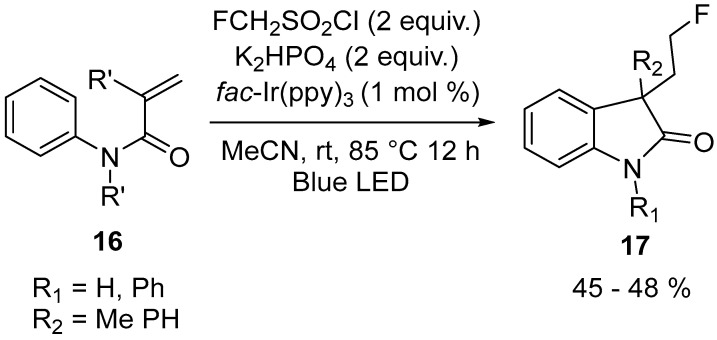
*N*-Methyl-*N*-phenylmethacrylamide cyclization using CH_2_FSO_2_Cl.

Fluoromethylation occurs in the presence of Ir-based photoredox catalysts (fac-[Ir(ppy)3] (ppy: 2-pyridylphenyl)), resulting in the formation of CH_2_F radicals. Fluoromethylsulfonyl chloride, as the source of the CH_2_F group, requires additional heating since the generation of CH_2_F radicals is a slower process, which is not observed in the case of CF_3_SO_2_Cl. *N*-Methyl-*N*-phenylmethacrylamide acts as the substrate that gives the target fluoromethylated 2-oxindoline [62,63] (Scheme 7).

**Scheme 8:**
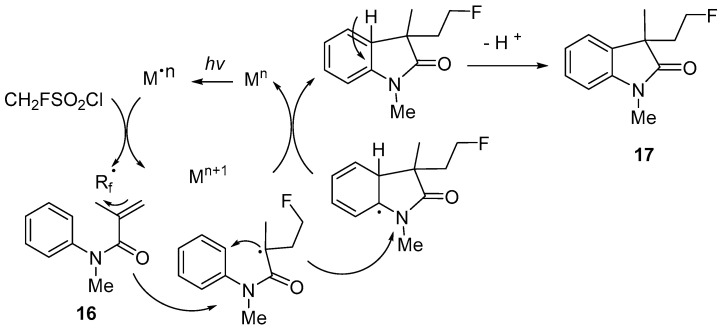
The proposed mechanism for *N*-Methyl-*N*-phenylmethacrylamide cyclization in the presence of CH_2_FSO_2_Cl.

The formation of the 2-oxindoline involves radical cyclization followed by the oxidation of the intermediate [62,63] (Scheme 8).

**Scheme 9:**
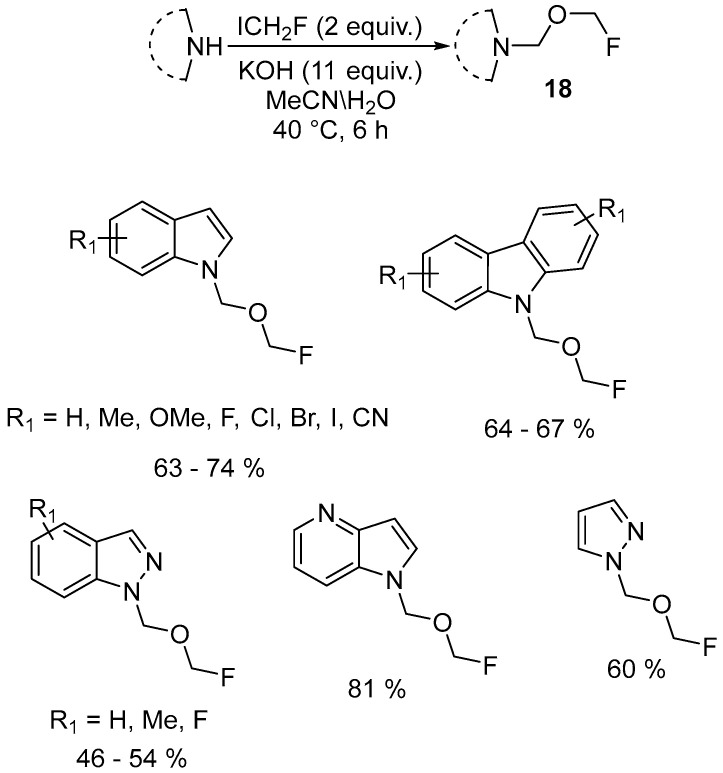
*N*-Fluoromethoxymethylation of aromatic heterocycles using ICH_2_F in the presence of KOH.

Fluoriodomethane is a commercially available, convenient-to-use (b.p. 53.4 °C) and effective reagent for the fluoromethylation of heterocyclic nucleophiles in the presence of bases. Fluoriodomethane allows for the *N*-fluoromethoxymethylation of a wide range of aromatic heterocycles in the presence of KOH. The reaction proceeds well with indoles, carbazoles, 1*H*-indazoles and pyrazoles with yields of 46–81% [64] (Scheme 9).

**Scheme 10:**
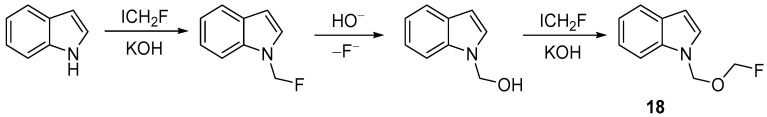
The proposed mechanism of *N*-fluoromethoxymethylation of aromatic heterocycles using ICH_2_F in the presence of KOH.

The process occurs with an excess of ICH_2_F (6 equiv.) and KOH (11 equiv.), MeCN and water in a volumetric ratio of 1:1. According to ^19^F NMR monitoring of the reaction, a control experiment and literature data, the reaction proceeds through fluoromethylation of the substrate to form an FCH_2_N intermediate, which is further hydrolyzed to form an *N*-hydroxymethylated derivative, which, in the last step, is electrophilically attacked by a second equivalent of ICH_2_F to form the final product **18** (Scheme 10).

A control experiment, consisting of the fluoromethylation of the previously obtained NCH_2_OH derivative, indirectly confirms this mechanism. Pyrrole, imidazole and benzotriazole do not react with ICH_2_F under the conditions considered [64].

**Scheme 11:**
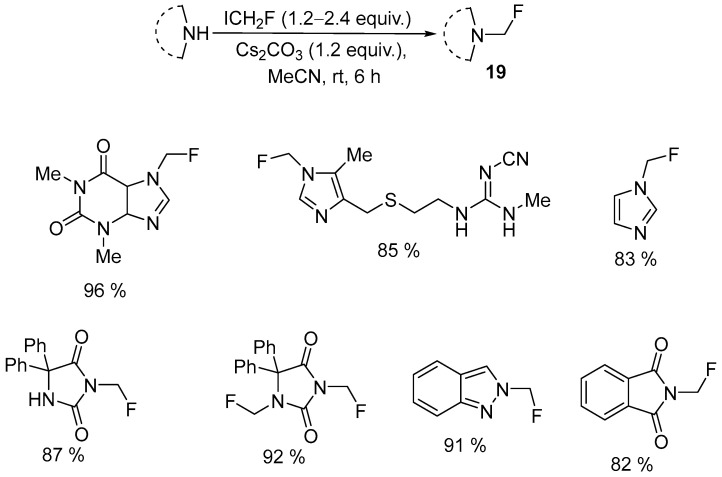
*N*-Fluoromethylation of *N*-heterocycles using ICH_2_F in the presence of Cs_2_CO_3._

Varying the bases and reaction medium makes it possible to carry out the fluoromethylation with ICH_2_F at different nucleophilic centers in the heterocyclic core or side substituents. In the presence of a slight excess of ICH_2_F (1.2 equiv) and cesium carbonate (1.2 equiv) in acetonitrile, the reaction proceeds quantitatively for 6 h for a wide range of *N*-heterocycles, including pharmaceutically important ones [65] (Scheme 11).

The method was successfully applied to the *N*-fluoromethylation of Theophylline. Cimetidine was also fluoromethylated in a similar manner without the guanidine moiety being reacted. Phenytoin reacts with both monofluoromethylation (excess of ICH_2_F 1.2 eq) and bisfluoromethylation of the hydantoin fragment (excess of 2.4 eq of ICH_2_F). Imidazole, indazole and phthalimide also react excellently, giving the products in a quantitative yield. Analytically pure heterocyclic derivatives were extracted from the reaction mixture with Et_2_O without silica gel chromatography [65]. *N*-Fluoromethylphthalimide was also obtained in the reaction of potassium phthalimide with fluoriodomethane in a MeCN medium. The yield is achieved at 71% by performing the reaction at 120 °C and increasing the pressure to 3.3 bar [66].

**Scheme 12:**
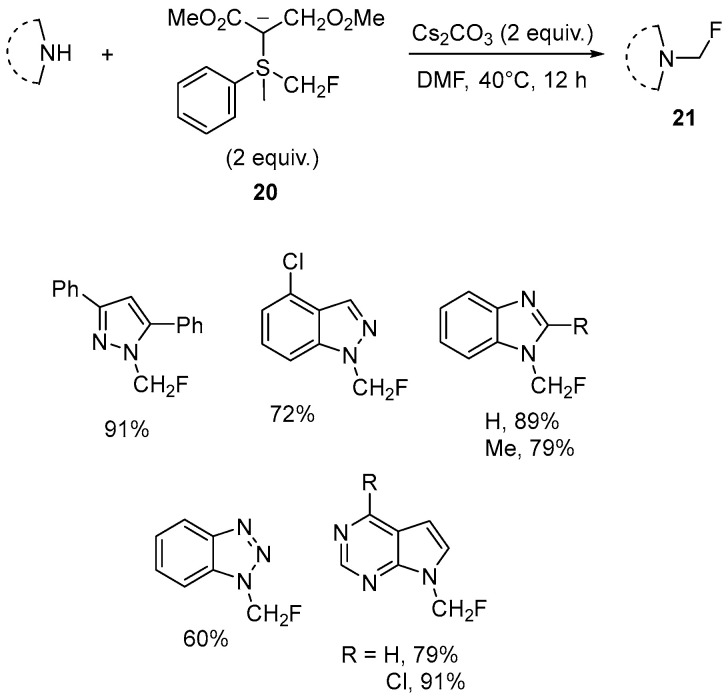
Electrophilic *N*-Fluoromethylation of heteroarenes using monofluoromethyl(aryl)sulfonium bis(carbomethoxy)methylide.

*N*-Heteroarenes can undergo *N*-fluoromethylation using electrophilic monofluoromethylating reagents—monofluoromethyl(aryl)sulfonium bis(carbomethoxy)methylide **20** under mild conditions [67] (Scheme 12).

Mechanistic studies with D-labeled reagents suggest that the reactions proceed through an electrophilic substitution pathway [67].

**Scheme 13:**
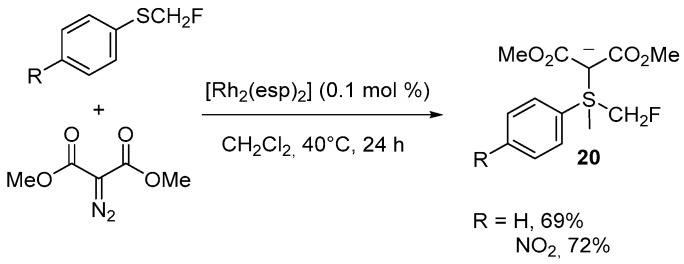
Synthesis of monofluoromethyl(aryl)sulfonium bis(carbomethoxy)methylide.

Monofluoromethyl(phenyl)sulfonium bis(carbomethoxy)methylide **20** was synthesized from chloromethylphenyl thioether and CsF in PEG-200/MeCN. The fluoromethylphenylthioether is further treated with dimethyldiazomalonate in the presence of [Rh_2_(esp)_2_] (esp = a,a,a′,a′-tetramethyl-1,3-benzenedipropionic acid) in dichloromethane [67] (Scheme 13).

Monofluoromethyl(aryl)sulfonium bis(carbomethoxy)methylide **20** is a solid substance that can be stored at room temperature for more than a month, which is very convenient to use, unlike gaseous reagents [67]. The synthesis of a number of similar monofluoromethylsulfonium ylides, derived from 5-diazo-2,2-dimethyl- 1,3-dioxane-4,6-dione, 2-diazo-1H-indene-1,3(2H)-dione and 3-diazo-1,1,1,5,5,5-hexafluoropentane-2,4-dione and used for the fluoromethylation of the same substrates that are presented in the Scheme 12 and some *N*-heterocyclic 1,3-dicarbonyl compounds, is described in the works [68,69].

**Scheme 14:**
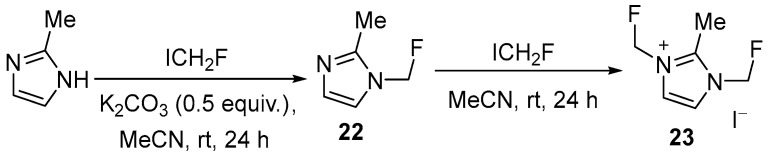
Bisfluoromethylation of 2-methylimidazolole.

2-Methyl imidazole also reacts with fluoriodomethane to form 1-fluoromethyl-2-methyl imidazole in MeCN in the presence of K_2_CO_3_ at room temperature. The reaction mixture was stirred overnight, and corresponding 1-fluoromethyl-2-methyl imidazole **22** was obtained with a yield of 63%. Adding another equivalent of iodofluoromethane leads to the formation of bisfluoromethyl-2-methylimidazolium iodide **23**, with a yield of 73% [66] (Scheme 14).

The melting point of bisfluoromethyl-2-methylimidazolium iodide is 252 °C [66].

**Scheme 15:**
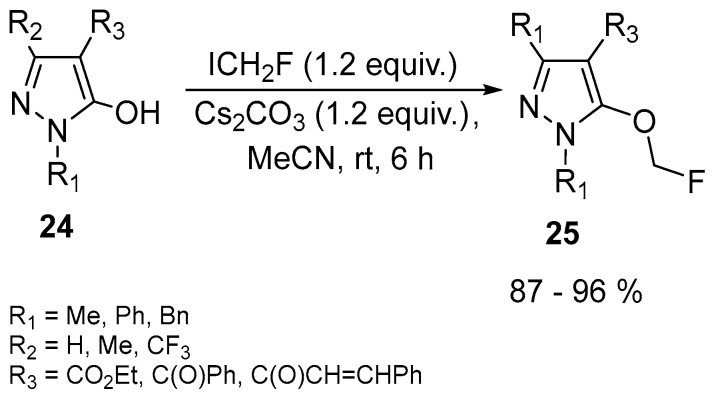
*O*-Fluoromethylation of *N*-substituted 5-hydroxypyrazoles **24** using ICH_2_F in the presence of Cs_2_CO_3_.

Fluoriodomethane allows direct *O*-fluoromethylation of *N*-substituted 5-hydroxypyrazoles **24** in quantitative yields [65] (Scheme 15).

In the pyrazole series, the substitution proceeds efficiently regardless of the type of substituents R_2_ and R_3_ (Scheme 15) [65].

**Scheme 16:**
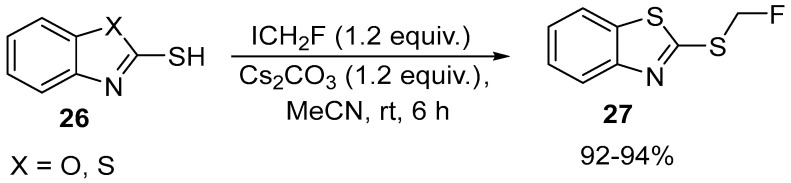
***S***-Fluoromethylation of 2-thio-benzo[d]oxazole and 2-thio-benzo[d]thiazole using ICH_2_F in the presence of Cs_2_CO_3_.

The reaction also occurs at the sulfur atom in 2-thio-substituted benzooxazole and benzothiazole **26** to give product **27** quantitatively [65] (Scheme 16).

**Scheme 17:**
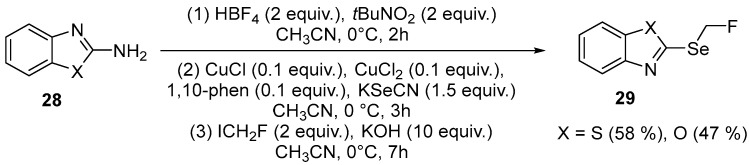
Synthesis of monofluoromethyl selenoethers using 2-(amimo)benzooxazole and 2-(amimo)benzothiazole.

2-(Amimo)benzooxazole and 2-(amimo)benzothiazole **28** can give similar Se-containg compounds. Monofluoromethyl selenoethers **29** are formed in the reaction of heterocyclic amines **28** and organoselenocyanates in several stages [70] (Scheme 17).

**Scheme 18:**
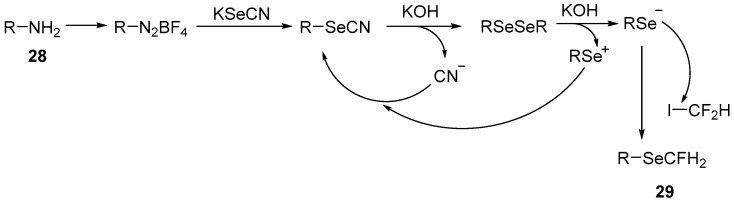
The proposed mechanism for the selenomonofluoromethylation of 2-(amimo)benzooxazole and 2-(amimo)benzothiazole.

The hetarylamine and *t*BuNO_2_ in the presence of HBF_4_ yield the intermediate, aryldiazonium tetrafluoroborate, which then proceeds in a Schiemann-type reaction with KSeCN. Dihetaryl diselenide RSeSeR is formed from RSeCN in the presence of KOH, which may also help promote nucleophilic attack of RSe-anion in ICH_2_F to form RSeCH_2_F. The resulting cyanate anion can also react with RSe^+^ to form another molecule of RSeCN [70] (Scheme 18).

Recently, the synthesis of unknown azidofluoromethane **30** was demonstrated for the first time [71]. The reaction of bromofluoromethane and sodium azide proceeds in an *N*-methyl-2-pyrrolidone (NMP) medium at room temperature with quantitative yield. Azidofluoromethane **30** appears to be an excellent reagent for the synthesis of *N*-fluoromethyl-substituted 1,2,3-triazoles **32**.

**Scheme 19:**
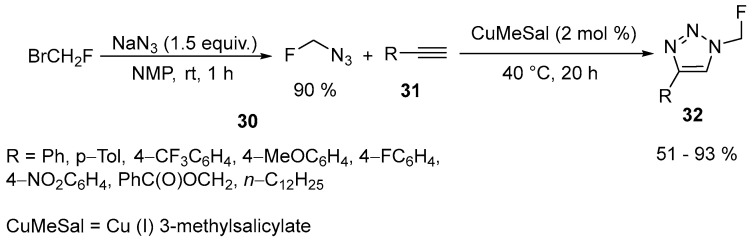
Synthesis of azidofluoromethane and 4-substituted 1,2,3-triazoles.

4-Substituted 1,2,3-triazoles **32** are formed in the reaction of terminal alkynes **30** and azidofluoromethane **31** in the presence of Cu(I) salt [71] (Scheme 19).

The reaction proceeds regioselectively with various phenylacetylenes or tetradec-1-yne in the presence of CuMeSal to form 4-substituted triazoles **32** [71].

**Scheme 20:**
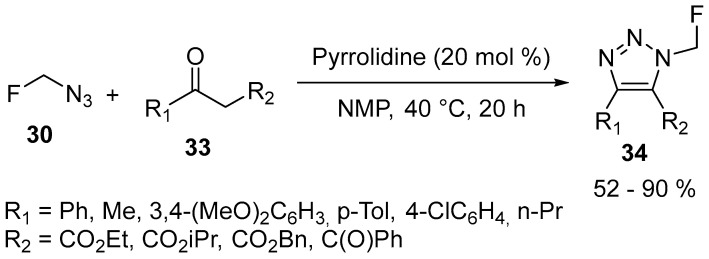
4,5-Disubstituted triazole formation through the reaction of azidofluoromethane using β-ketoesters or 1,3-diones.

4,5-Disubstituted triazoles are formed through a similar reaction of azidofluoromethane with β-ketoesters or 1,3-diones in the presence of pyrrolidine in good to high yields [71] (Scheme 20).

The formation of *N*-fluoromethyl-substituted 1,2,3-triazoles has not been described in the literature, with the exception of the synthesis of related 1-fluoromethyl-substituted benzotriazoles, which were obtained through electrophilic substitution reactions between monofluoromethylchloride [72] or sulfoximines [73] and the corresponding benzotriazoles.

**Scheme 21:**
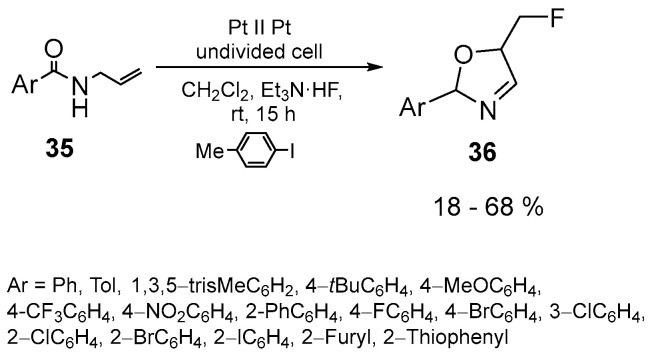
Electrochemical synthesis of 5-fluoromethyl-2-oxazolines.

5-Fluoromethyl-2-oxazolines **36** were obtained using the fluorocyclization of *N*-allylcarboxamides **35** in yields of up to 68% under electrochemical oxidation conditions. 4-Methyliodobenzene or 4-*tert*-butyliodobenzene acted as a mediator, and the source of fluorine was Et_3_N·5HF [74] (Scheme 21).

**Scheme 22:**
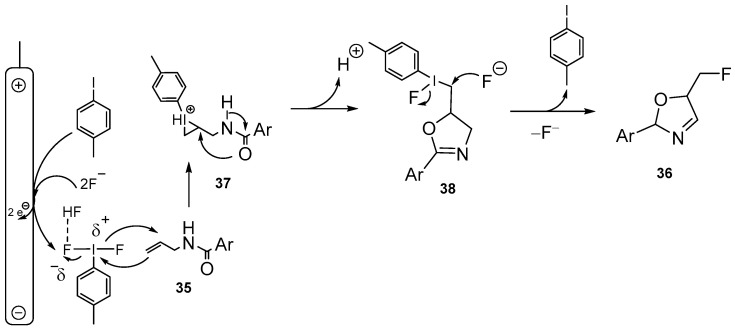
The proposed mechanism for the electrochemical synthesis of 5-fluoromethyl-2-oxazolines.

The proposed mechanism for the electrosynthesis of 5-fluoromethyl-2-oxazolines **36** includes the stage of anodic oxidation of iodorene, resulting in the formation of (difuoroiodo)arene. Next, the iodine (III) atom undergoes a nucleophilic attack through the double bond of the allyl amide **35** to form the iodonium intermediate **37**, followed by intramolecular ring-opening via a carbonyl oxygen atom to form 5-(*λ*^3^-iodanyl)methyloxazoline **38**. The last stage is S*_N_*2 substitution with a fluoride anion that yields the target product [74] (Scheme 22).

A similar synthesis was carried out in a flow reactor in the presence of Et_3_N·7HF and CH_2_Cl_2_ (1:1) and 4-iodotoluene as an oxidation mediator. The yields of the target 5-fluoromethyl-2-oxazolines **36** reach 77%. The method is suitable for preparing gram-scale quantities (the productivity of the process is 100–312 mg/h) [75].

**Scheme 23:**
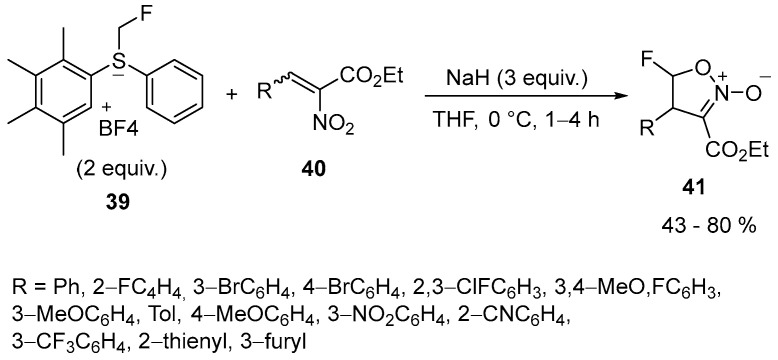
Synthesis of C5-monofluorinated isoxazoline N-oxides.

**Scheme 24:**
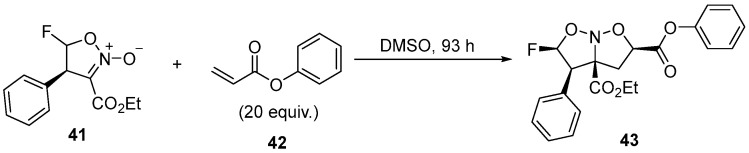
[3 + 2]-Dipolar cycloaddition of isoxazoline-N-oxides with phenylacrylate.

2-Nitroacrylates **39** and monofluoromethyl sulfides **40** are excellent starting compounds for the synthesis of C5-monofluorinated isoxazoline *N*-oxides **41**. The reaction was shown for the first time in the work [76]. The reaction does not require special conditions and expensive components: tetrahydrofuran is an excellent reaction solvent, and sodium hydride gives the best yield of sulfur fluoromethylide **39** in situ. The corresponding isoxazoline-N-oxides were prepared in moderate to very good yields, and the compounds were stable even when in contact with silica gel during isolation (Scheme 23):

The resulting isoxazoline-*N*-oxides **41** can undergo [3 + 2]-dipolar cycloaddition with alkenes as a 1,3-dipole. The reaction proceeds well with terminal alkenes activated by electron-withdrawing groups, for example, dipolarophiles such as phenylacrylate **42** [76] (Scheme 24).

Earlier, *N*-monofluoromethylation of azoles with ClCH_2_F was shown under similar conditions [72].

### 2.3. Monofluoromethylation of Six-Membered N-heterocycles

**Scheme 25:**
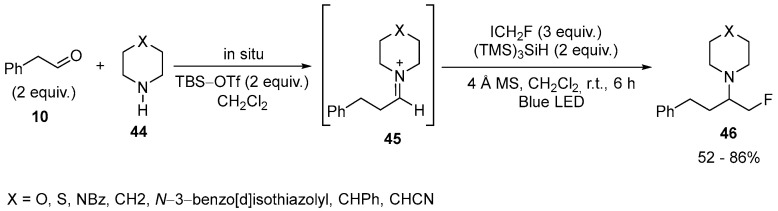
*N*-Monofluoromethylation of saturated *N*-containing heterocycles using aldehydes and fluoroiodomethane.

Saturated *N*-containing heterocycles **44** can undergo monofluoromethylation with 2-phenylacetaldehyde **10** and fluoroiodomethane under blue LED radical initiation conditions in the presence of (TMS)_3_SiH. The method makes it possible to synthesize tertiary α-fluoromethyl heterocyclic derivatives **46** that are difficult to obtain otherwise (Scheme 25) [27].

The reaction involves the formation of the iminium cation **45** in situ, which is an acceptor of the CH_2_F radical, followed by a reaction with (TMS)_3_SiH for the HAT process, giving the target product. To assist the formation of the active iminium species, *t*Budimethylsilyltrifluoromethanesulfonate (TBS-OTf) was used as a convenient Lewis acid additive [27].

**Scheme 26:**
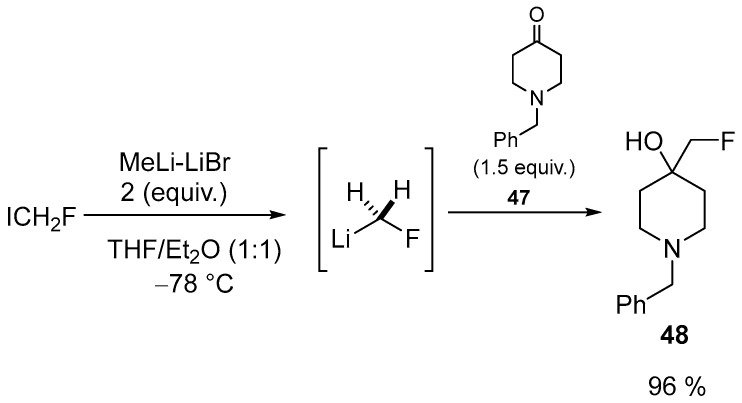
4-Monofluoromethylation of 1-benzylpiperidin-4-one with lithium fluorocarbenoid.

Piperidin-4-one **47** undergoes direct nucleophilic fluoromethylation with lithium fluorocarbenoid (LiCH_2_F) obtained from fluoriodomethane [77] (Scheme 26). The corresponding fluoroaminoalcohol **48** was obtained in quantitative yield. The reaction proceeds similarly to that shown in Scheme 1.

**Scheme 27:**
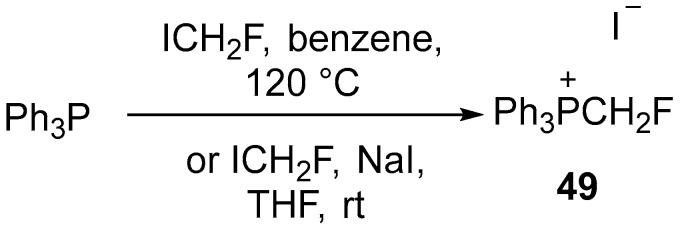
The synthesis of (fluoromethyl)triphenylphosphonium iodide.

Fluoriodomethane or fluorobromomethane are precursors in the synthesis of (fluoromethyl)triphenylphosphonium iodide **49** through the reaction of stoichiometric amounts of Ph_3_P and ICH_2_F or BrCH_2_F as a source of CH_2_F [78] (Scheme 27).

**Scheme 28:**
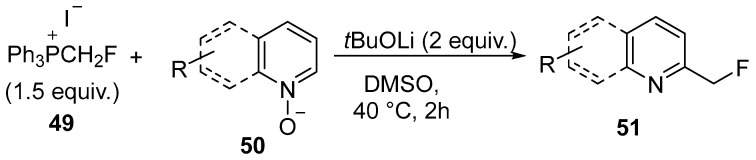
2-Fluoromethylation of pyridine-*N*-oxides.

Fluoromethylene phosphonium ylide **49** is used in the direct C2-fluoromethylation of a wide range of pyridine-*N*-oxides [78] (Scheme 28).

**Scheme 29:**
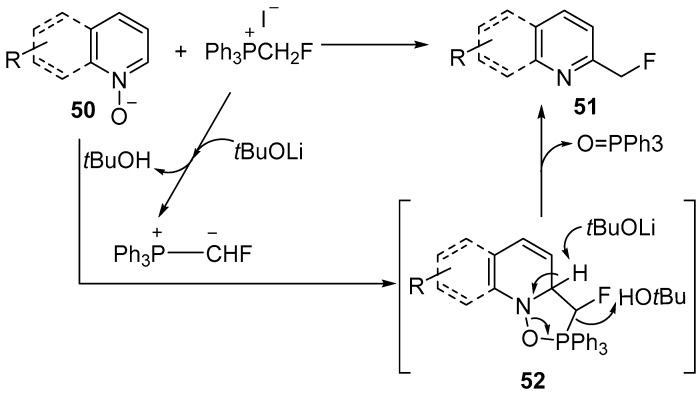
The proposed mechanism for the 2-fluoromethylation of pyridine-*N*-oxides.

The reaction proceeds in DMSO with a 1.5-fold excess of (fluoromethyl)triphenylphosphonium iodide **49** and a two-fold excess of *t*BuOLi at 40 °C for 2 h. Pyridine *N*-oxides **50** containing electron-donating or electron-withdrawing groups in different positions of the pyridine ring were used as substrates. Fluoromethylation of *N*-oxides of 2,2′-bipyridine, quinoline and phenanthridine were also studied (yields 20 to 79%). However, 2,6-disubstituted pyridine *N*-oxides and 2-substituted quinoline *N*-oxides do not react under the considered conditions [78]. The proposed mechanism involves the reaction of (fluoromethyl)triphenylphosphonium iodide **49** with *t*BuOLi to produce fluoromethylene phosphonium ylide. Next, the annulation reaction of heteroaryl-N-oxides and the intermediate ylide **52** occurs, followed by aromatization to form fluoromethylated products [78] (Scheme 29).

**Scheme 30:**
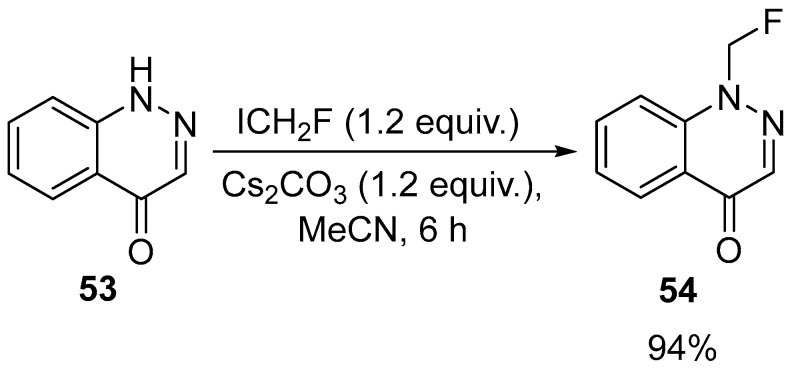
*N*-Fluoromethylation of 4(1*H*)-cinnolinone with ICH_2_F in the presence of Cs_2_CO_3._

In the presence of cesium carbonate, similar to the *N*-fluoromethylation of imidazoles and pyrazoles [65], the reaction of 4(1*H*)-cinnolinone **53** and ICH_2_F proceeds excellently (yield 94%) [65] (Scheme 30).

**Scheme 31:**
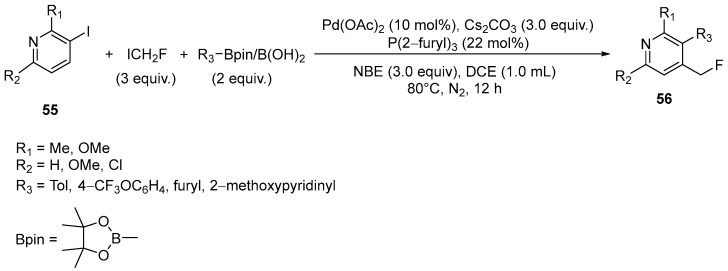
Synthesis of *ortho*-fluoromethylated pyridines in the presence of arylboronates.

Fluoriodomethane, the industrial raw material, is the source of the CH_2_F group, which shows excellent tolerance to different functional groups. In the presence of the Pd/norbornene C-H catalytic system, aryl fluoromethylation proceeds well [79] (Scheme 31).

The aryl iodide undergoes a reaction with Pd(0), insertion of a norbornene molecule (NBE), followed by ortho-CH activation to form norbornyl palladacycles, which react with ICH_2_F and a nucleophile to form the final fluoromethylated product. Optimized conditions assume the presence of DCE (0.2 M), Pd(OAc)_2_ (10 mol%), tris(2-furyl)phosphine (P(2-furyl)_3_, 22 mol%) as the ligand, NBE (3.0 equiv.) as the catalytic mediator, and Cs_2_CO_3_ (3.0 equiv.). The reaction occurs most efficiently at 80 °C for 12 h. The reaction could be complicated due to direct cross-coupling between arylboronates and ICH_2_F; however, pinacol arylboronic esters can act as coupling partners in this case. In the synthesis of unsymmetrical orthofluoromethylated biaryls, yields are 45–86%. *Ortho*-substituted aryl boronic esters can perform a similar function. In this case, yields are 45–76%, respectively. *N*-Heterocyclicboronic pinacol esters were used to prepare furan and pyridine derivatives [79].

**Scheme 32:**
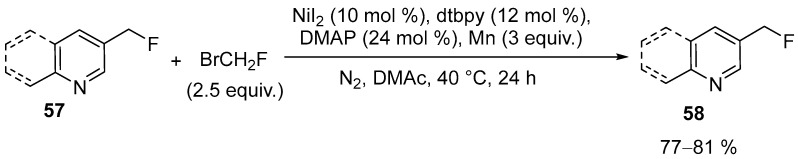
NiI_2_-Catalyzed CH–monofluoroalkylation of pyridine and quinoline.

Pyridine and quinoline also undergo direct monofluoromethylation with BrCH_2_F in the presence of NiI_2_ and dtbpy (4,4-Di-tert-butyl-2,2-dipyridine) and DMAP (4-dimethylaminopyridine) as ligands through reductive cross-coupling in the presence of metallic Mn [80] (Scheme 32).

This strategy provides an effective method for the synthesis of monofluoromethylated molecules for drug discovery [80].

**Scheme 33:**
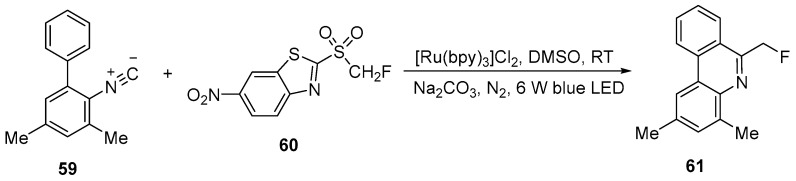
The synthesis of 6-fluoromethylphenanthridine through the reaction of 2-((fluoromethyl)sulfonyl)-6-nitrobenzothiazole with 2-isocyano(1,1′-diphenyl).

The synthesis of 6-fluoromethylphenanthridine was carried out through the reaction of monofluoromethyl 2-benzo[d]thiazolyl sulfone and 2-isocyano-3,5-dimethyl-1,1′-biphenyl. The reaction proceeds as radical fluoroalkylation of isocyanides through homolytic cleavage of the C-S bond in the presence of a photo-red/ox catalyst under blue LED. Monofluoromethylsulfone is the source of the fluoroalkyl radical. The generation of a monofluoromethyl radical from monofluoromethyl-2-benzo[d]thiazolyl sulfone is difficult under the given conditions compared to trifluoromethyl or difluoromethyl analogs due to the smaller electron-withdrawing effect of the monofluoromethyl group. However, the introduction of the NO_2_ group into the 2-position of benzo-[*d*]thiazolyl fragment significantly increases the monofluoromethylation reaction activity (Scheme 33) [81].

**Scheme 34:**
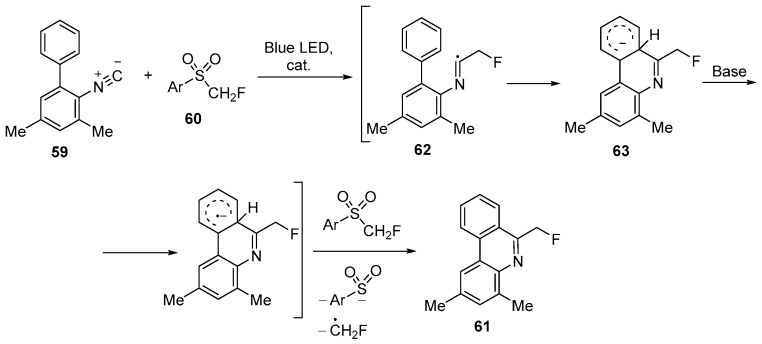
Proposed mechanism of the assembling of 6-fluoromethylphenanthridine.

The proposed mechanism involves a photo-red\ox cycle of the catalyst, resulting in a one-electron transfer at the monofluoromethylsulfone with the formation of a sulfinate ion and a monofluoromethyl radical that combines with the isocyanide **59** to form an imidoyl radical **62**. The latter undergoes intramolecular radical cyclization to form intermediate product **63**, which gives the final product **61** in the presence of Na_2_CO_3_ [81] (Scheme 34).

**Scheme 35:**
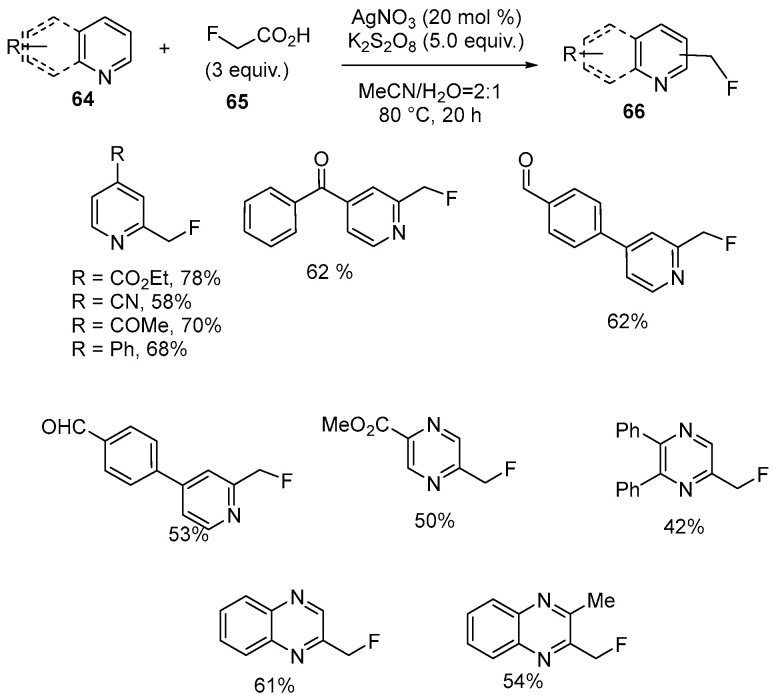
C–H–monofluoromethylation of pyridines, pyrazines and quinoxaline.

Stable α-monofluorocarboxylic acids **64** allow the direct C–H-monofluoroalkylation of pyridines, pyrazines and quinoxalines **65** to form compounds **66** [82] (Scheme 35).

*N*-Heteroarenes show high efficiency in the fluoroalkylation reaction, providing access to functionalized fluoroalkyl-substituted structures for the synthesis of drugs. The reaction is catalyzed using silver salts and probably proceeds through a radical mechanism [82].

**Scheme 36:**
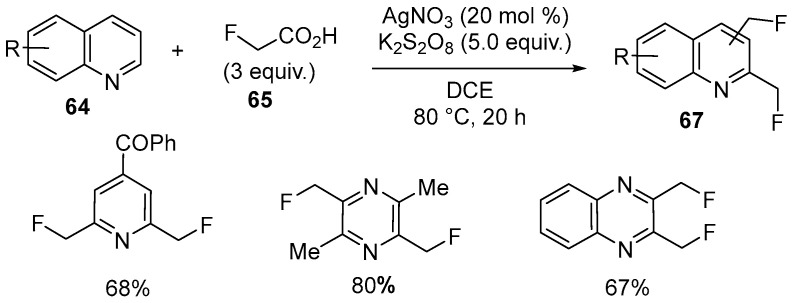
C–H–bis(monofluoro)methylation of pyridines, pyrazines and quinoxalines.

The formation of bis-monofluoroalkylated pyridine, pyrazine and quinoxaline **68** occurs more efficiently when replacing MeCN with DCE and using five equivalents of α-monofluorocarboxylic acid **65** [82] (Scheme 36).

**Scheme 37:**
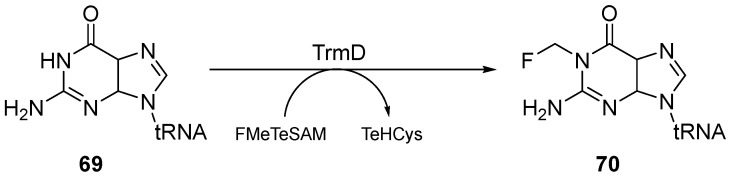
Fluoromethylation of tRNA guanine N37 using FMeTeSAM.

The synthesis of Te-adenosyl-L-(fluoromethyl)homotellurocysteine (FMeTeSAM) **68**, a novel biologically active enzymatic fluoromethylating agent, was recently described. FMeTeSAM is structurally and chemically related to the versatile cellular agent S-adenosyl-L-methionine (SAM) and allows for the thermally mediated transfer of fluoromethyl groups to nitrogen, sulfur and certain C-nucleophiles. FMeTeSAM is used for the synthesis of fluorine derivatives of guanine **70** from **69**, natural compounds with anticancer properties [83] (Scheme 37):

FMeTeSAM is a promising reagent in the field of biocatalytic reactions and the synthesis of natural compounds and their analogs, exhibiting high chemoselectivity and regioselectivity [83].

**Scheme 38:**
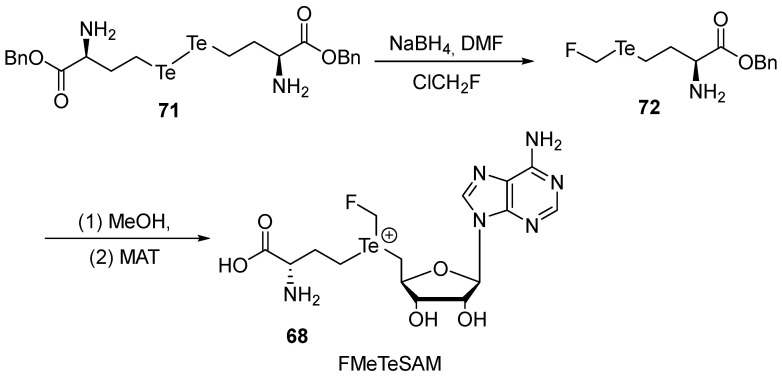
Synthesis of FMeTeSAM.

The synthesis of FMeTeSAM **68** includes six sequential steps starting from L-homoserine. The two final stages are shown in Scheme 38. Ditelluride **71** is reduced with NaBH_4_ and fluoromethylated using ClCH_2_F by bubbling gas through the reaction mass. The last stage is a process in the presence of ATP and MAT. Methionine adenosyl transferase (MAT) catalyzes the synthesis of S-adenosylmethionine, giving the desired product **68** [83]:

Fluorinated *S*-adenosylmethionine could potentially provide a similar reagent for enzyme-catalyzed fluoromethylation of *N*-heterocycles [84].

**Scheme 39:**
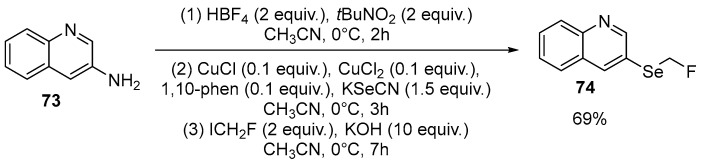
Synthesis of monofluoromethyl selenoesters from quinoline-3-amine.

Quinoline-3-amine **73** yields the corresponding monofluoromethyl selenoester **74** in the reaction of selenocyanate with ICH_2_F. The synthesis is a one-pot, multi-step procedure. The method is general for a wide range of amino group-containing substrates (Scheme 39) [70]:

The reaction is the first example of the synthesis of RSeCH_2_F compounds. The proposed mechanism for a similar reaction is similar to the one presented in Scheme 18.

The data concerning *N*- and *C*-fluoromethylation of nitrogen-containing heterocycles are summarized in Table 2.

## 3. Conclusions

Selective monofluoromethylation is one of the least studied methods in fluoroalkylation compared to trifluoro- and difluoromethylation. This review shows the existence of many methods for implementing this reaction with the formation of biologically significant fluorinated *N*-heterocycles. The reactions proceed regioselectively and in high yields. Bis(fluoromethylation) with a threefold excess of the fluorinating agent has been shown for pyridines, pyrazines and quinoxalines. In modern organofluorine chemistry, fluoriodomethane is the main, effective and universal agent for the introduction of the CH_2_F group into a wide variety of heterocyclic cores. Fluoriodomethane is tolerant to most functional groups, making it a convenient reagent. Its physical characteristics, availability and the possibility of carrying out electrophilic, nucleophilic and radical methodologies make it a very important and promising reagent for the introduction of the CH_2_F group. However, other fluoromethylating agents (BrCH_2_F, ClCH_2_F, TBAF and FCH_2_SO_2_Cl, monofluoromethyl(aryl)sulfoniummethylides, monofluoromethyl sulfides, (fluoromethyl)triphenylphosphonium iodide, 2-fluoroacetic acid) do not lose their relevance. Azidofluoromethane, recently obtained, is an excellent building block for the synthesis of monofluorinated 1,2,3-triazoles.

Modern monofluoromethylation methods almost eliminate the need to use ozone-depleting substances. The development of the synthesis of promising fluoroalkylation reagents in the field of biocatalytic reactions and the synthesis of natural compounds and their analogs is of great importance.

## Figures and Tables

**Table 1:** Approved or investigational drugs containing of the CH_2_F group.

Structure	Therapeutic Use	Approval Status
Carmegliptin 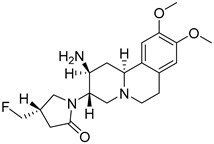	Inhibitor of dipeptidyl peptidase 4 (DPP-4), with hypoglycemic activity for monotherapy or in combination with other oral antihyperglycemic agents for the treatment of Type 2 diabetes	Investigational drug; second phase of clinical trials [43,44];
Fluticasone furoate 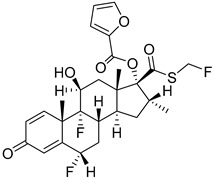	Corticosteroid with potent anti-inflammatory, anti-allergic action	2007
Sevoflurane 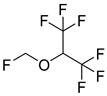	General anesthetic	1995
Sirpefenicol 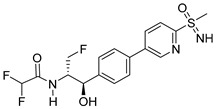	Antibacterial agent	Investigational veterinary drug;
Norcholestenol fluoromethyl 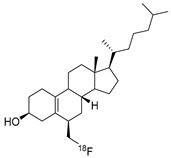	Fluorinated analog of the scintiscanning/SPECT agent for PET imaging	Investigational PET imaging agent [45];
Imlunestant 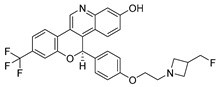	Orally active ER-α degrader, antiestrogen and antineoplastic agent for the treatment of ER-positive (ER+) breast cancers;	Investigational drug [46,47,48,49,50]; third phase of clinical trials;
Fipravirimat 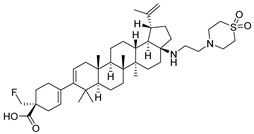	Anti-HIV/AIDS; maturation inhibitors;	Investigational drug [51];
^18^F-FMISO or fluoromisonidazole 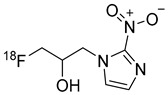	Radiopharmaceutical for PET imaging of hypoxia	Investigational drug [52,53];
Mefway 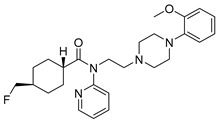	Serotonin 5-HT1A receptor antagonist; PET radiotracer	Investigational drug [54,55];
Florfenicol 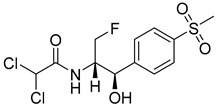	Antibiotic	1996
Cefluprenam 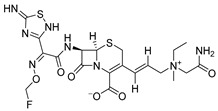	Anti-bacterial agent of broad spectrum; fourth generation cephalosporin; Highly effective against bacterial pneumonia;	1997
Afloqualone 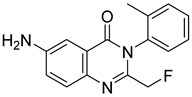	Sedative and muscle-relaxant	Investigational drug [56];
Fluorocholine 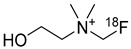	Oncologic PET tracer	Investigational drug [57];

**Table 2:** *N*- and *C*-fluoromethylation of three-, five- and six-membered *N*-heterocycles.

Scheme №	Product	Substrate	CH_2_F-Source, Conditions	Ref.
	Three-Membered *N*-heterocycles			
1	α-Fluoromethyl-α-CF_3_ aziridine	Trifluoroacetimidoyl chloride (TFAIC)	ICH_2_F; MeLi-LiBr THF, −78 °C, 1 h	[58]
2	α-Fluoroaziridine	*N*-tosyl-diphenyl ketimine	ICH_2_F; LiN(iPr)Cy,	[59]
Five-Membered *N*-heterocycles				
3	3-Fluoromethylated *N*-benzylpyrrolidines	*N*-Benzylmaleimide	ICH_2_F; (Me_3_Si)_3_SiH, blue LED	[60]
4	1-(1-Fluoro-4-phenylbutan-2-yl)pyrrolidine	Pyrrolidine	ICH_2_F; 2-phenylacetaldehyde, (Me_3_Si)_3_SiH, blue LED	[27]
6	3-Fluoromethylated indolines	Spiro-epoxyoxindolines	TBAF	[61]
7	3-(2-Fluoroethyl)indolin-2-ones	*N*-Methyl-*N*-phenylmethacrylamides	FCH_2_SO_2_Cl; K_2_HPO_4_ fac-Ir(ppy)_3_ (1 mol%)	[62,63]
9	*N*-Fluoromethoxymethylated indoles, carbazoles, 1*H*-indazoles and pyrazoles	Indoles, carbazoles, 1*H*-indazoles and pyrazoles	ICH_2_F; KOH MeCN/H_2_O 40 °C, 6 h	[64]
11	N-fluoromethylated Theophylline, Cimetidine, Phenytoin (hydantoin), imidazole, indazoles, phthalimide	Theophylline, Cimetidine, Phenytoin (hydantoin), Imidazole, Indazole, phthalimide	ICH_2_F, Cs_2_CO_3_. Theophylline, Cimetidine, Phenytoin (hydantoin), Imidazole, Indazole, phthalimide	[65]
12	*N*-Fluoromethylated pyrazole, indazole, benzoimidazole, benzotriazole, 7*H*-pyrrolo[2,3-d]pyrimidine	1*H*-pyrazole, 1*H*-indazole, benzoimidazole, benzotriazole, 7*H*-pyrrolo[2,3-d]pyrimidine	Monofluoromethyl(aryl)sulfonium bis(carbomethoxy)methylide; Cs_2_CO_3_, DMF; 40 °C, 12 h	[67,68,69]
14	1-Fluoromethyl-2-methyl imidazole; bis(fluoromethyl)-2-methylimidazolium iodide	2-Methyl imidazole	ICH_2_F; K_2_CO_3_, MeCN, rt	[66]
15	*O*-fluoromethylated *N*-substituted 5-hydroxypyrazoles	*N*-substituted 5-hydroxypyrazoles	ICH_2_F, Cs_2_CO_3_, MeCN 6 h, rt	[65]
16	2-((Fluoromethyl)thio)benzo[d]thiazole, 2-((Fluoromethyl)thio)benzo[d]thiazole	2-thio-benzooxazole and 2-thio-benzothiazole	ICH_2_F, Cs_2_CO_3_, MeCN 6 h, rt	[65]
17	2-((fluoromethyl)selanyl)benzooxazole, 2-((fluoromethyl)selanyl) benzothiazole	2-(Amimo)benzooxazole, 2-(Amimo)benzothiazole	ICH_2_F; KSeCN, KOH	[70]
19	4-Substituted *N*-fluoromethyl-1,2,3-triazoles	Terminal alkynes	Azidofluoromethane; NMP, Cu (I) 3-methylsalicylate, rt, 1 h	[71]
20	4,5-Disubstituted *N*-fluoromethyl-1,2,3-triazole	β-Ketoesters or 1,3-Diones	Azidofluoromethane; pyrrolidine, 40 °C, 20 h	[71]
21	5-Fluoromethyl-2-oxazolines	*N*-Allylcarboxamides	Et_3_N·5HF; Electrochemical conditions; 4-Methyliodobenzene, 4-*tert*-butyliodobenzene; CH_2_Cl_2_, rt, 15 h	[74,75]
23	5-Monofluorinated isoxazoline *N*-oxides	2-Nitroacrylates	Monofluoromethyl sulfides; NaH, 40 °C, 20 h	[76]
Six-Membered *N*-heterocycles				
25	1-(1-Fluoro-4-phenylbutan-2-yl) pyperidine, piperazines, morpholine, thiomorpholine	2-phenylacetaldehyde Pyperidine, piperazines, morpholine, thiomorpholine	ICH_2_F; *t*Budimethylsilyltrifluoromethanesulfonate (TBS-OTf) (TMS)_3_SiH; 4 Å MS, CH_2_Cl_2_, r.t., 6 h Blue LED	[27]
26	1-Benzyl-4-(fluoromethyl)piperidin-4-ol	Piperidin-4-one	ICH_2_F; MeLi-LiBr THF, −78 °C, 1 h	[77]
28	C2-fluoromethylated pyridines, 2,2′-bipyridine, quinoline and phenanthridine-*N*-oxides	Pyridines, 2,2′-bipyridine, quinoline and phenanthridine-*N*-oxides	(fluoromethyl)triphenylphosphonium iodide	[78]
30	1-(Fluoromethyl)cinnolin-4(1*H*)-one	4(1*H*)-cinnolinone	ICH_2_F, Cs_2_CO_3_, MeCN 6 h, rt	[65]
31	*Ortho*-fluoromethylated pyridines	3-Iodo-2,5-disabstituted pyridines	ICH_2_F; Pinacol arylboronic esters (RBpin) Pd(OAc)_2_ (10 mol%), Cs_2_CO_3_ (3.0 equiv.) P(2-furyl)_3_ (22 mol%)	[79]
32	3-(Fluoromethyl)pyridine; 3-(Fluoromethyl)quinoline	Pyridine, quinoline	BrCH_2_F; NiI_2_; dtbpy (4,4-Di-tert-butyl-2,2-dipyridine); DMAP (4-dimethylaminopyridine); N_2_, DMAc, 40 °C, 24 h	[80]
33	6-Fluoromethylphenanthridine	2-Isocyano-3,5-dimethyl-1,1′-biphenyl	2-((fluoromethyl)sulfonyl)-6-nitrobenzothiazole; [Ru(bpy)_3_]Cl_2_, DMSO, rt, Na_2_CO_3_, N_2_, Blue LED	[81]
35	2-Monofluoromethylated pyridines, pyrazines and quinoxalines	Pyridines, pyrazines and quinoxalines	2-fluoroacetic acid; AgNO_3_, K_2_S_2_O_8_, MeCN/H_2_O = 2:1 80 °C, 20 h	[82]
36	2,6-Bis(monofluoromethyl)pyridine; 2,5-Bis(monofluoromethyl) pyrazines; 2,3-Bis(monofluoromethyl)quinoxalines	Pyridines, pyrazines and quinoxalines	2-fluoroacetic acid; AgNO_3_, K_2_S_2_O_8_, DCE, 80 °C, 20 h	[82]
39	3-((Fluoromethyl)selanyl)quinoline	Quinoline-3-amine	ICH_2_F; HBF_4_/*t*BuCN CH_3_CN, 0 °C, 2 h; CuCl/CuCl_2_/1,10-phen/ KSeCN CH3CN, 0 °C, 3 h; KOH CH_3_CN, 0 °C, 7 h	[70]

## Data Availability

Not applicable
